# Supplementary material for: Therapeutic potential of fisetin in hepatic steatosis: Insights into autophagy pathway regulation and endoplasmic reticulum stress alleviation in high-fat diet-fed mice
Source: PLoS One. 2025 May 22;20(5):e0322335. doi: 10.1371/journal.pone.0322335 (PMC12097571; doi:10.1371/journal.pone.0322335)
Supplement: S1 Table — (DOCX) [file pone.0322335.s001.docx]

**Supplementary Table 1**

Primers sequences used in this study.

| **Reverse** | **Forward** | **Primer** |
| --- | --- | --- |
| **5ʹ- ATGGAGCCACCGATCCACA -3ʹ** | **5ʹ-CATCCGTAAAGACCTCTATGCCAAC -3ʹ** | **β-Actin** |
| **5ʹ- GGAGGAGACACGAAGCAGACT -3ʹ** | **5ʹ- ACAACACTGACCTGGACACTT -3ʹ** | **GRP78** |
| **5ʹ- TTTCATGTCATAAAGTTGTAGGTTAGG-3ʹ** | **5ʹ-CAACGTGGCAGCCTTACA -3ʹ** | **eIF2A** |
| **5ʹ- ACTGTGGCGTTAGAGATCGT -3ʹ** | **5ʹ- CGGCTGGTCGTCAACCTAT -3ʹ** | **ATF4** |
| **5ʹ- GGTGCCCCCAATTTCATCT -3ʹ** | **5ʹ- CCACCACACCTGAAAGCAGAA -3ʹ** | **CHOP** |
| **5ʹ- AAGGTGGCATTGAAGACATT -3ʹ** | **5ʹ- AGCGGGAGTATAGTGAGTTT -3ʹ** | **BECN1** |
| **5ʹ- ACGAAGGCTGGGTTCATGC -3ʹ** | **5ʹ- GTTTGTGGCTCTGAATGACCA -3ʹ** | **MTOR** |
| **5ʹ- TTCTTGGAGAGTGCTCAGGC-3ʹ** | **5ʹ-TCCCTACACACCTTCTCCCC -3ʹ** | **ULK1** |
| **5ʹ- TTCTGGGGTAGTGGGTGTCA -3ʹ** | **5ʹ- GAATGTGGGGGAGAGTGTGG -3ʹ** | **SQSTM1/p62** |
| **5ʹ- TCCAAGGAAGAGCTGAACTTGA -3ʹ** | **5ʹ- AAGGCACACCCCTGAAATGG -3ʹ** | **ATG5** |
