# Supplementary material for: Therapeutic potential of fisetin in hepatic steatosis: Insights into autophagy pathway regulation and endoplasmic reticulum stress alleviation in high-fat diet-fed mice
Source: PLoS One. 2025 May 22;20(5):e0322335. doi: 10.1371/journal.pone.0322335 (PMC12097571; doi:10.1371/journal.pone.0322335)
Supplement: Attachment — Submitted filename: fistin manuscript.docx [file pone.0322335.s002.docx]

**Therapeutic potential of fisetin in hepatic steatosis: insights into autophagy pathway regulation and endoplasmic reticulum stress alleviation in high-fat diet-fed mice**

Mahboobe Sattari ^1^, Mohammad Esmaeil Shahaboddin ^2^, Maryam Akhavan Taheri ^3^

Ehsan Khalili ^1^, Ozra Tabatabaei-Malazy ^4,5^, Golnaz Goodarzi ^6^, Sadra Samavarchi Tehrani ^7^

Reza Meshkani ^1^, Ghodratollah Panahi ^1^*^✉^

^1^ Department of Clinical Biochemistry, Faculty of Medicine, Tehran University of Medical Sciences, Tehran, Iran.

^2^ Institute for Basic Sciences, Research Center for Biochemistry and Nutrition in Metabolic Diseases, Kashan University of Medical Sciences, Kashan, Iran.

^3^ Institute for Basic Sciences, Anatomical Sciences Research Center, Kashan University of Medical Sciences, Kashan, Iran.

^4^ Non-Communicable Diseases Research Center, Endocrinology and Metabolism Population Sciences Institute, Tehran University of Medical Sciences, Tehran, Iran.

^5^ Endocrinology and Metabolism Research Center, Endocrinology and Metabolism Clinical Sciences Institute, Tehran University of Medical Sciences, Tehran, Iran.

^6^ Department of Pathobiology and Laboratory Sciences, School of Medicine, North Khorasan University of Medical Sciences, Bojnurd, Iran.

^7^ Endocrine Research Center, Institute of Endocrinology and Metabolism, Iran University of Medical Science, Tehran, Iran.

^✉^email: pshahriyar@gmail.com; ghpanahi@sina.tums.ac.ir

**ABSTRACT**

Non-alcoholic fatty liver disease (NAFLD) is a common condition with limited FDA-approved treatments due to its complex pathogenesis. Metabolic stress-induced lipotoxicity triggers the unfolded protein response, leading to the development of NAFLD through inflammation and apoptosis. Moreover, metabolic dysregulation compromises autophagic capacity, impairing effective ERphagy and lipophagy in the liver.

Fisetin (FSN), a flavonoid present in various fruits and vegetables, has demonstrated the ability to regulate the processes mentioned above and possesses a range of biological properties. In this study using a high-fat diet-induced NAFLD mouse model, treatment with FSN at a dosage of 80 mg/kg per day for eight weeks resulted in reduced hepatic lipid accumulation. This effect was mediated by modulating ER stress through enhancing autophagic activity, as indicated by decreased expression of GRP78, elf2a, ATF4, and CHOP genes, along with increased AMPK phosphorylation, decreased mTOR expression, and elevated levels of ULK1, ATG5, and Beclin1. Additionally, there was an increase in the LCII/LC3I ratio and a reduction in p62 levels in hepatic tissue. Our findings suggest that FSN exerts its effects by activating the AMPK/mTOR signaling pathway and its downstream targets, underscoring its potential therapeutic advantages in managing NAFLD by targeting autophagy and ER stress pathways.

**Keywords:** Fisetin; Autophagy; Endoplasmic Reticulum Stress; Nonalcoholic Fatty Liver Disease; hydroxy Chloroquine.

**INTRODUCTION**

Non-alcoholic fatty liver disease (NAFLD), which is caused by hepatic lipid overload, is the most globally prevalent chronic liver disease. The intricate pathogenic mechanism has posed challenges in the development of drugs for NAFLD, as no related medications have been approved by FDA. As such, the first line of treatment for NAFLD remains lifestyle modification (1, 2). However, because long-term weight loss diets are particularly difficult to stick to (3), there is considerable interest in identifying agents affecting key pathogenic pathways in order to treat or prevent this illness.

NAFLD is often associated with obesity, insulin resistance (IR), and metabolic syndrome (4). Free fatty acids (FFAs) are widely mobilized from adipocytes to the liver as a result of IR. This process increases the amount of β-oxidation of FFAs in the mitochondria, which causes electrons to leak out of the electron transport chain. This can result in mitochondrial dysfunction and oxidative stress (5). At high concentrations, reactive oxygen species cause oxidative modifications to cellular compounds and causes a build-up of damaged macromolecules, resulting in liver injury (6). In stress conditions like excessive FFA levels and lipotoxicity, improperly folded and unfolded proteins amass in the endoplasmic reticulum (ER) lumen, triggering the unfolded protein response (UPR) to reestablish ER equilibrium, as shown in NAFLD patients (7, 8). ER stress caused by metabolic stress is associated with changes in membrane and lipid biosynthesis, inflammation, and apoptosis, adding to the transition from simple steatosis to steatohepatitis (9). Autophagy, as an adaptive mechanism in cellular damage, plays a crucial role in the removal and engulfment of damaged organelles and misfolded proteins(10). Also, a specific type of autophagy, known as lipophagy, involves the breakdown of lipid droplets in the cytosol. It has been documented that NAFLD patients exhibit decreased autophagy (11, 12). The molecular mechanism underlying autophagy dysfunction in NAFLD involves both short-term regulations, where the pathway is inhibited by the mTOR complex activated due to overfeeding, and long-term regulation, where hyperinsulinemia contributes to its inhibition (13). Furthermore, high-fat diet-induced changes in membrane lipid composition exacerbate the reduction of autophagy by causing defects in autophagosome-lysosome fusion (14). The compromised autophagic capacity fails to effectively remove damaged organelles (15, 16). Since autophagy defects contribute to a vicious cycle in various aspects of NAFLD pathogenesis, the induction of autophagy may represent a promising target for therapeutic interventions (13, 17).

Herbal compounds, particularly flavonoids, are gaining popularity as non-toxic and preventive medicinal treatments. Flavonoids exhibit beneficial effects against NAFLD by preventing hepatosteatosis, reducing calorie intake, body weight, and visceral fat deposition, while acting as potent antioxidants to mitigate inflammation and insulin resistance. Furthermore, in NAFLD patients, flavonoids improve blood pressure, glucose tolerance, insulin sensitivity, dyslipidemia, and adiponectin levels (18). Fisetin (FSN), a flavonoid present in vegetables and fruits, has demonstrated numerous biological properties, including anti-invasive, anti-angiogenic, anti-inflammatory, antioxidant, and regulation of cell metabolism and autophagy in various conditions such as cancer, diabetes, cardiovascular and neurological diseases (19, 20). In this study we investigate how FSN affects NAFLD by examining autophagy and ER stress, key processes linked to NAFLD advancement. To investigate the role of autophagy in NAFLD, we used hydroxychloroquine (HCQ) a lysosomotropic drug that inhibits autophagy and macroautophagy. HCQ prevents lysosomal degradation by increasing the pH within lysosomes and we aimed to understand how the suppression of autophagy affects the progression and pathology of NAFL (21). The study's insights provide information about FSN's therapeutic potential for NAFLD and its complications, offering promise for the development of safe and effective treatments.

**MATERIALS AND METHODS**

**Drugs and Chemicals**

FSN, with a (HPLC) purity of ≥ 98% and CAS No. 528-48-3 (Batch No. TF20220119), was acquired from Henan Tianfu Chemical Co. Ltd, located in Henan, China. HCQ with CAS No. 747-36-4 (Batch No. HCS0040299) was provided by Tehran Chemical Ph. Raw Material Co. in Tehran, Iran. The primary antibodies utilized in western blot analysis comprised LC3B (#2775 Cell Signaling), p62 (sc-10117, Santa Cruz, CA), p-AMPKα1/2 (sc-33524, Santa Cruz, CA), AMPKα1/2 (sc-74461, Santa Cruz, CA), and β-Actin (sc-517582, Santa Cruz, CA). The secondary antibody employed in western blot analysis were the mouse anti-rabbit IgG-HRP antibody (sc-2357, Santa Cruz, CA) and m-IgGκBP-HRP (sc-516102, Santa Cruz, CA).

**Animals**

Male C57BL/6J mice weighing 18–20 g at 7–8 weeks of age were acquired from the Iranian Pasteur Institute. They were housed in a controlled environment with a constant temperature of 23 - 25 ^◦^C, humidity ranging from 50–60%, and a standard 12-hour light/12-hour dark cycle. The mice had *ad libitum* access to water and pathogen-free food. Each cage was provided with enrichment (plastic tube and shredded paper). The cages were checked daily and cleaned once a week. All animal experiments were conducted according to guidelines approved by the Research Ethics Committees of Laboratory Animals at Tehran University of Medical Science (IR.TUMS.AEC.1401.126).

**Experimental Protocol**

Following a two-week adaptation period, one of two diets was given to mice at random: the standard chow diet (SCD) (10.2% fat and 71.5% carbohydrates; D12450J; Research Diets) (n=8), and the high-fat diet (HFD) (60% calories from fat; D12492; Research Diets) (n=40) to induce NAFLD model (22). After 16 weeks, five groups were randomly selected from the HFD group (n=8): HFD; HFD+Vehicle (V); HFD+FSN; HFD+HCQ and HFD+FSN+HCQ. FSN was dissolved in DMSO and tween80, then diluted in 0.9% normal saline for daily gavage administration to mice (80 mg/kg) alongside the HFD for 8 weeks. HCQ in buffered aqueous solution was injected intraperitoneally (i.p) twice a week (50 mg/kg, 0.1 mL/10g body weight) for 8 weeks (Fig. 1a). Dosage adjustments were made according to the weekly body weight. Mice diet intake was tracked daily, and body weight was recorded every week. Caloric intake was calculated as the weight (g) of food consumed times the diet's calories (kcal) (23). The %weight gain / caloric intake (gkcal) x 100 formula is used to compute the food efficiency ratio (24).

**Metabolic Tolerance Tests**

Metabolic tolerance was assessed at the end of the treatment period through oral glucose tolerance test (OGTT) and insulin tolerance test (ITT) (25). For OGTT, blood glucose levels were measured after a 10-hour nocturnal fasting period. Subsequently, an α-D-glucose solution (10%) was administered (2 g/kg) by gavage. Blood glucose levels were monitored at 15, 30, 60, and 120-minute intervals. For ITT, blood glucose levels were measured after a 6-hour morning fasting period. Then, an insulin solution (0.25 IU/ml) was i.p administered (0.75 IU/Kg). Blood glucose levels were monitored at 15, 30, 60, and 90-minute intervals. The measurements utilized a glucometer (On Call Plus®) to analyze a blood drop from the mice's tail tip.

**Sampling**

The mice were allowed to fast for ten hours at the conclusion of the treatment period before being decapitated and killed by CO_2_ asphyxiation. After being drawn into a tube, about 0.8 mL of blood was left to stand at room temperature for 20 minutes. Following a 10-minute centrifugation at 3000 rpm for the blood sample, different biochemical parameters were examined in the supernatant. Subsequently, the body was dissected, and the liver tissues were removed, weighed, and divided into multiple portions for different experimental objectives. One part was put into a tube that had been filled with a formalin solution (%4). For subsequent analysis in Western blot and qRT-PCR studies, the remaining tissues were frozen in liquid nitrogen and placed in a refrigerator at -80°C.

**Biochemical Parameters Analysis**

Serum levels of triglyceride (TG), total cholesterol (TC), LDL-C, HDL-C, alanine aminotransferase (AST) and aspartate aminotransferase (ALT) were determined with a kit that can be purchased commercially (Zeist Chem Diagnostics. Tehran, Iran) in CL2000i Autoanalyzer.

**Liver Histological Analysis**

Liver tissue specimens were preserved for a minimum of twenty-four hours in paraformaldehyde. Following a graded series of ethanol dehydration, the specimens were cleaned in xylene and embedded in paraffin wax. Using a rotary microtome, tissue blocks were sectioned at a 5 µm thickness. Hematoxylin and eosin were used to stain the sections. Histological features were viewed using an Olympus light microscope (Olympus, Tokyo, Japan) by a qualified pathologist. The liver histopathological changes were evaluated by calculating the NAFLD activity score (NAS), which considered four criteria: steatosis, ballooned hepatocytes, lobular inflammation, and fibrosis stage (26).

**RNA Extraction and Quantitative Real-Time PCR**

The Favorgen Tissue Total RNA Mini Kit (Ping Tung, Taiwan) was utilized to extract total RNA from fresh frozen liver tissues. The quality and concentration of extracted RNA were assessed using a NanoDrop^TM^ device (Thermo Scientific NanoDrop OneC). A cDNA synthesis kit was used to reverse transcribe 1000 ng of extracted RNA into cDNA (yekta tajhiz azma, Tehran, Iran). The hepatic mRNA levels of key molecules involved in UPR regulation including Glucose-Regulated Protein 78 (GRP78), activating transcription factor 4 (ATF4), Eukaryotic initiation factor 2A (eIF2A) and C/EBP homologous protein (CHOP), as well as molecules involved in the regulation of autophagy, such as the mechanistic target of rapamycin kinase (mTOR), unc-51-like autophagy-activating kinases 1 (ULK1), ATG6/Beclin-1 (BECN1), autophagy related 5 (ATG5), and Sequestosome 1 (p62/SQSTM1) were measured using YTA® Universal qPCR Master Mix (Yekta Tajhiz Azma, Tehran, Iran) on a real-time PCR cycler (Qiagen Rotor-Gene Rt Pcr Machine). Table 1 in Appendix S1 Table contains a list of the primers that were used in this investigation to measure the mRNA expression levels in Real-Time PCR.

**SDS-PAGE and Western-Blot Analysis**

Lysis buffer was used to homogenize mouse liver tissues. The Bradford method was used to measure the protein concentrations in the supernatant after centrifugation (27). The reference protein was bovine serum albumin. Protein lysate in equal amounts was directly loaded onto a 10%–12% SDS-PAGE and then placed onto a PVDF membrane for protein blotting. The membranes were incubated with the designated primary antibodies for 16 h at 4°C after being blocked for 75 min at room temperature using 2% non-fat dry milk (w/v) in Tris-buffered saline with 0.1% Tween 20 detergent (TBS-T). After that, membranes were probed for 75 min at room temperature using either m-IgGκBP-HRP or mouse anti-rabbit IgG-HRP secondary antibodies. Each gel lane contained an aliquot of the pooled standard sample. The ECL advanced reagents were used to identify the immuno-reactive bands. β-actin functioned as a loading regulator. With Image J, a densitometric analysis was carried out.

**Statistical Analyses**

The data is shown as mean ± S.E.M. One-way ANOVA was used to identify statistical differences, and Tukey's multiple comparison test was then performed. P values less than 0.05 were deemed statistically significant. *P < 0.05, **P < 0.01, ***P < 0.001, and ****P < 0.0001 denote statistically significant differences. To conduct statistical analyses, Prism 9 (GraphPad, San Diego, CA) was used.

**RESULTS**

**Fisetin alleviates HFD-induced obesity and food efficiency ratio in mice**

We fed HFD to mice for 16 weeks in order to induce NAFLD (Fig 1b). As depicted in Fig 1c, from week 4 until the end of the experiment, the HFD-fed mice's body weight increased significantly more than that of the SCD group. This was due to the augmented mean caloric intake in HFD group in comparison to SCD group. After 8 weeks of drug treatment, there were no variations found in the caloric intake analyzed between groups consuming HFD, except for the HCQ group (20%) (Fig 1d), which exhibited a significant weight loss compared to other groups. Despite the same caloric intake, FSN slightly and FSN+HCQ significantly hindered the food efficiency ratio in contrast to HFD mice (Fig 1e).

**Fig 1. Fisetin alleviates HFD-induced obesity and food efficiency ratio in mice.** (a) Schematic diagram of the experimental procedure. (b) A mouse fed SCD on the left and HFD on the right with their weight gain after 16 weeks. (c) Change of body weight during a 24-week study period. (d) The average Caloric intake and (e) food efficiency ratio during the 8th week of treatment (n = 8). The information is displayed as mean ± S.E.M. *P < 0.05, **P < 0.01, ***P < 0.001, and ****P < 0.0001.

**Fisetin enhances glucose homeostasis in HFD-fed mice**

Comparing HFD-fed mice to SCD-fed mice, the former had higher levels of fasting plasma glucose (FPG), both after 6 and 10 hours, while mice in treatment groups had a reduction that became significant for HCQ and FSN+HCQ groups in the longer time of fasting (Figs 2a,b). SCD mice were demonstrated to have a glucose tolerance by the OGTT, while mice fed on HFD appeared to have a decreased glucose tolerance. The glucose tolerance of mice treated with FSN and FSN+HCQ was significantly improved (Fig 2c). The area under the curve (AUC) ascertained the above results (Fig 2d). During ITT, SCD and HFD reached under 50% of basal concentration of glucose about by 30 and 60 minutes, respectively after insulin injection. The treated groups showed this reduction in a time closer to the SCD group (Fig 2e). The AUC elucidated that insulin sensitivity improved statistically after FSN, HCQ and FSN+HCQ treatment (Figs 2f, g).

**Fig 2. Fisetin enhances glucose homeostasis in HFD-fed mice.** the average level of fasting plasma glucose (FPG) in mice after (a) 6 hours and (b) 10 hours of fasting. (c) Oral glucose tolerance test (OGTT) and (d) area under the curve (AUC) of blood glucose level. (e) Mean percent reduction of blood glucose from baseline on time points after insulin injection. (f) Insulin tolerance test (ITT) and (g) AUC of blood glucose level (n = 5). The information is displayed as mean ± S.E.M. *P < 0.05, **P < 0.01, ***P < 0.001, and ****P < 0.0001.

**Fisetin preserve lipid homeostasis in HFD-fed mice**

Serum TG was significantly reduced by all three types of treatment compared to HFD (Fig 3a). FSN treatment didn’t reduce serum TC (Fig 3b), but significantly reduced LDL-C (Fig 3c). Treatment with FSN+HCQ significantly reduced serum TC (Fig 3b), accompanied by a decrease in LDL-C (Fig 3c), while the proportion of HDL-C increased (Fig 3d).

**Fig 3. Fisetin preserve lipid homeostasis in HFD-fed mice.** Serum levels of lipid biochemical parameters in mice. (a) Triglyceride (TG), (b) Total cholesterol (TC), (c) LDL-C and (d) HDL-C/TC (n=5). The information is displayed as mean ± S.E.M. *P < 0.05, **P < 0.01, ***P < 0.001, and ****P < 0.0001.

**Fisetin alleviates NAFLD development in HFD-fed mice**

Grossly, the HFD group showed fatty liver-like changes with hepatomegaly, paler coloration, and greasy appearance. In contrast, the normal group had dark brown/red livers, while the FSN and FSN+HCQ groups regained partly this color (Fig 4a). H&E staining indicated that treatment with FSN, HCQ and FSN+HCQ significantly alleviated NAS in the livers of mice induced by HFD (Figs 4a,b). This cumulative score was mainly due to the suppressed individual components of steatosis and inflammation (Figs 4c-e). Additionally, the HFD group was observed to have considerably increased liver weight/body weight (LW/BW) (Fig 4f), hepatic TG and TC contents (Figs 4g,h), as well as, serum liver enzymes (AST and ALT) (Figs 4i,j) compared with SCD groups. In comparison to the untreated group, these parameters were significantly lower in the FSN+HCQ-treated group. Although FSN did not have a significant effect on the relative weight of the liver, it improved all of other mentioned parameters. According to the findings, an 8-week FSN and FSN+HCQ treatment protected liver from lipid accumulation under HFD.

**Fig 4. Fisetin alleviates NAFLD development in HFD-fed mice.** (a) The morphology of livers (upper) and representative histological pictures of sections stained with Hematoxylin and Eosin. in the middle (100× magnification) and the lower (400× magnification). Small and large fat vacuoles are abundantly visible in hepatocytes in the HFD and vehicle groups (arrows). In the vehicle group, accumulation of lymphoid cells is shown (arrowhead). In the FSN and HCQ groups, fat vacuoles can be seen in some hepatocytes, but their reduction is visible. The FSN+HCQ group is very similar to the SCD group. Only a few numbers of small lipid droplets are observed in some cells. (b) The NAFLD activity, (c) steatosis, (d) ballooning and (e) lobular inflammation scores. (f) The ratio of liver weight per body weight (LW/BW) (g/g*100). Hepatic tissue (g) TG and (h) TC content. Hepatic biochemical parameters in serum; (i) Aspartate aminotransferase (AST) and (j) Alanine aminotransferase (ALT) (n=5). The information is displayed as mean ± S.E.M. *P < 0.05, **P < 0.01, ***P < 0.001, and ****P < 0.0001.

**Fisetin inhibits ER stress in liver of HFD-fed mice**

HFD stimulates ER stress as evidenced by the significant increases in the mRNA levels of GRP78, eIF2A, ATF4, and CHOP. These effects were significantly attenuated upon administration of FSN and FSN+HCQ (Figs 5a–d). These results demonstrated how FSN and FSN+HCQ suppress ER stress, which in turn improves the development of NAFLD.

**Fig 5. Fisetin inhibits ER stress in liver of HFD-fed mice.** The relative mRNA level of (a) GRP78, (b) eIF2A, (c) ATF4 and (d) CHOP genes was quantified using real-time PCR (n=5). The information is displayed as mean ± S.E.M. *P < 0.05, **P < 0.01, ***P < 0.001, and ****P < 0.0001.

**Fisetin induces autophagy, which lowers the accumulation of hepatic lipids in HFD-fed mice**

The process by which LC3-I becomes LC3-II by conjugating with phosphatidylethanolamine (PE) is the gold standard marker for autophagosome formation (28). Since p62 is an autophagy substrate, it is also essential. In order to assess the relationship between FSN and autophagy flux, we used western blot analysis of these variables. Western blot revealed that the levels of LC3II/LC3I ratio were significantly diminished while p62 was accumulated in liver tissue lysates of C57BL/6J mice fed on HFD (Figs 6a,b). After FSN treatment, LC3II/LC3I was more prominent, while p62 expression was reduced, suggesting that FSN could trigger autophagy. However, either enhanced synthesis or decreased degradation could be the cause of elevated LC3II/LC3I levels. Therefore, it may be necessary to assess autophagic flux using the lysosomal inhibitor HCQ. When HFD mice were administered HCQ, there was a slight increase in the LC3II/LC3I ratio. The combined administration of FSN along with HCQ in the sixth group led to a significant enhancement of this effect, suggesting the induction of autophagy up to the final phase and its subsequent cessation (Figs 6a,b). But about p62, it didn’t accumulate simultaneously with a decrease in autophagy flux in the two last groups.

In order to assess autophagic activity, we also measured the levels of AMPK phosphorylation and mTOR gene expression. p-AMPK promotes autophagy by inhibiting mTOR, which in turn inhibits autophagy when activated. The expression of p-AMPK was significantly downregulated in liver of HFD-fed mice (Fig 6c). This was followed by an increased transcriptional level of mTORc-1 (Fig 6d), as inhibitor of autophagic agents, as well as decreased levels of Beclin-1 (Figs 6e) which is essential for carrying out autophagy, along with decreased levels of SQSTM1/p62, ULK1 and ATG5 (Figs 6 f-h). These alterations notably prevented after treatment with FSN, HCQ and FSN+HCQ for 8 weeks.

**Fig 6. Fisetin induces autophagy, which lowers the accumulation of hepatic lipids in HFD-fed mice.** The western blot results for proteins involved in autophagy; (a) LC3II/LC3I, (b) p62, and (c) AMPK phosphorylation in mice (n=3). The expression levels of autophagy-related genes; (d) MTOR, (e) BECN1, (f) SQSTM1/p62, (g) ULK1, and (h) ATG5 (n=5). The information is displayed as mean ± S.E.M. *P < 0.05, **P < 0.01, ***P < 0.001, and ****P < 0.0001.

Altogether, our study unveils certain aspects of how FSN functions in NAFLD, as illustrated in Fig 7.

**Fig 7. Fisetin functions in NAFLD.** This schematic demonstrates that HFD-induced metabolic syndrome results in heightened ER stress severity, leading to hepatic lipid accumulation, while autophagy is impaired and insufficient under these conditions. Fisetin enhances autophagic flux through the AMPK/mTOR pathway, thereby alleviating ER stress and reducing lipid accumulation.

**DISCUSSION**

In recent years, several plant compounds have demonstrated potential in ameliorating- NAFLD. For instance, luteolin, resveratrol and quercetin has been shown to attenuate NAFLD (29, 30). Research conducted on animals has also reported the therapeutic potential of FSN in NAFLD. When male C57BL/6 mice were given an HFD, administering FSN orally decreased hepatic inflammation and lipid accumulation caused by the HFD by inhibiting the activation of TNF-α/RIPK3 axis (31). In NAFLD rat model induced by an HFD, oral administration of FSN improved HNF4-α/lipin-1 signaling, lowered oxidative stress, and suppressed reactive oxygen species-induced TXNIP induction and PARP-1 activation (32).

It has been reported that the regulatory actions of FSN in mediating ER stress regulate the advancement of chronic illness (33). Additionally, studies have shown that inducing autophagy can modulate ER stress (34). It has not yet been thoroughly explored, though, whether FSN can control these pathways—particularly their possible interplay—in order to successfully treat NAFLD. Thus, using a mouse model of HFD-induced NAFLD, we set out to examine any potential ameliorative effects of the bioactive flavonol FSN.

In the context of this study, providing an HFD for 16 weeks allowed for the successful establishment of a C57BL/6J model of NAFLD. To elucidate the underlying molecular mechanisms, we tested the effects of FSN supplementation on hepatic steatosis, along with ER stress and autophagy alterations. We also used HCQ as an autophagic inhibitor, which is an immune system modulator, in combination with FSN and individually.

Here, we present novel data demonstrating that eight weeks of FSN treatment in HFD-fed mice not only suppressed lipid accumulation in hepatic tissue, but also improved the underlying pathological features of NAFLD including modulating ER stress through autophagy activation. FSN therefore has the ability to improve NAFLD brought on by obesity. Unexpectedly, an important finding of this experiment was that HCQ coadministration could reinforce the beneficial effects of FSN on hepatic lipid accumulation which may offer fresh perspectives on medical care of metabolic complications.

In the present study, we observed that eight weeks of treatment with FSN or FSN+HCQ decreased the mice weight gain, by reducing the food efficiency ratio without changing the caloric intake. In line with our results, in various studies, FSN administration has prevented body weight gain (32, 35). The HCQ group suffered from cachexia, which was in part the result of insufficient food intake. It has been shown that inhibiting mitophagy as a part of the general process of autophagy prevents HFD-induced weight gain by inhibiting intestinal lipid absorption (36). Also, it has been seen in Fan et al.'s study that chronic treatment with CQ can cause muscle atrophy in mice (37).

In this study, treatment by FSN and more effectively by FSN+HCQ improved serum lipid profile and hepatic fat content. Several other studies showed the same results as our study regarding the effect of FSN on circulating and hepatic lipids (32, 38).

It has been demonstrated that ER stress inducers can cause lipid accumulation in hepatocytes (39, 40), suggesting that ER stress mediates liver steatosis (41). HFD can also throw off the protein homeostasis of ER equilibrium, which can trigger the UPR and ER stress development (42). According to some studies, FFAs induced ER stress in hepatocytes, as shown by the ER stress proteins, resulting in the lipids build-up and lipotoxicity (43, 44). In a different study, it was demonstrated that HFD induced ER stress in the mice livers based on the significantly higher expression of GRP78 and CHOP (45). Furthermore, we demonstrated that the HFD group exhibited increased mRNA expression of the UPR pathway elements, such as GRP78, elf2a, ATF4, and CHOP, in contrast to the SCD group. Multiple intracellular stress pathways may be activated as a result of ER stress. These paths can induce or hasten the development of dyslipidemia, insulin resistance (IR), inflammation, and apoptosis—all of which are involved in the pathophysiology of NAFLD (46, 47). Here, we discovered that the mice livers given FSN and FSN+HCQ had lower levels of GRP78 and CHOP gene expression than the controls. In particular, we observed reduced mRNA expression of the transcription factors elf2 and ATF4, which suggests suppression of the PERK-elf2a-ATF4 arm in the liver. This pathway holds significant importance as it controls steatosis and lipogenesis by upregulating FAS, ACC, and SCD-1 protein expression levels as well as the mRNA expression levels of SREBP-1c and ChREBP (48). Dai et al., consistently showed that FSN mainly contributed to GRP78-mediated ER stress inhibition in an HFD-induced murine model with NAFLD and hepatocytes, which inhibited reactive oxygen species generation and mitochondrial dysfunction (45). These results suggested the ER stress-suppressive properties of FSN, which in turn help prevent the development of NAFLD.

There are two main ways to control lipid droplet catabolism in the liver: lipolysis and lipophagy. Lipid droplets are specifically taken up by the autophagosome and transferred to the lysosome where lipase breaks them down during the lipophagy process (49). The impairment of this is intimately related with the development of NAFLD to non-alcoholic steatohepatitis (NASH) (50). In this investigation, the genes and proteins implicated in the three primary stages of autophagy—initiation (AMPK, mTORC1, and ULK1), nucleation (Beclin1), and elongation/maturation (Atg5 and p62 proteins, as well as the LC3BII/I ratio)—were assessed in order to assess the role of autophagy in the impact of FSN on liver steatosis.

Negative regulation of mTORC1 is one of the ways that activated p-AMPK has been demonstrated to promote autophagy (51). Since mTORC1 signaling is the primary gate regulating autophagy, autophagy is triggered when mTORC1 is inhibited by stressors or starvation. (52). We found that FSN and FSN+HCQ treatment enhanced AMPK phosphorylation and decreased mTOR mRNA expression in compare to control. In line with these results, Liou et al showed in a study that FSN treatment increased the expression of p-AMPK in the liver of C57BL/6 male mice compared to the HFD group (53). Jung et al indicated that FSN inhibits mTORC1 signaling via suppression of Akt activation in preadipocytes (54). In this study, FSN group showed increments mRNA levels of genes involved in autophagy including ULK1, ATG5, and Beclin1 which can be due to mTOR inactivation. Interestingly, some studies have suggested that FSN inhibits mTORc1 activity through direct binding (55). Collectively, our data showed that FSN applies its effects by inducing AMPK/mTOR signaling and its downstream mediators.

FSN upregulated the expression of LC3B at the protein level, along with an increase of LC3II/LC3I in liver tissue of mice. In addition, hepatic p62 protein levels lowered in the FSN-treated mice, indicating activation of autophagic flux by this natural product. Ding et al showed that FSN promotes mitophagy through upregulating LC3‐II and downregulating p62 in cardiac microvascular endothelial cells (CMECs) of rats with sepsis‐associated encephalopathy (56). Moreover, in a study by Zhang et al, the administration of FSN (80 mg/kg) provoked LC3 puncta accumulation and promoted the expression of p62 in acetaminophen-damaged liver tissue of Mice (57). Our study found that autophagy was inhibited in liver of HFD-mice, leading to increased lipid accumulation in the liver. While, FSN effectively reversed these alterations. These findings suggest that FSN improves intracellular lipid metabolism, which is at least in part result from the promotion of autophagic flux.

A more interesting finding in our study is that the decrease in p62 is despite the increase in its gene transcription induced by FSN. It seems that a part of a larger decrease in p62 is compensated and so covered by the increase in its production. Therefore, the net result is only a slight decrease in p62 protein, while we observed an increase in autophagy activity in this group by the LC3II/LC3I ratio as an autophagy hallmark. Kim et al have also shown an increase in relative mRNA level of SQSTM1/p62 expression with FSN in T4 cells and neurons (58). The concentration of this protein is actually subject to intricate modifications at the transcription and post-transcriptional levels. As a result, the measurement of p62 content as a proxy for autophagy activity remains debatable and is largely susceptible to interpretation errors. This matter has also been seen in the case of other autophagy inducers such as resveratrol, which exert their effect on both levels of protein synthesis and degradation (59). In order to prevent misunderstandings, it could be instructive to employ pharmaceuticals that block either the input or output current. Unexpectedly, HCQ co-treatment did not cause an increase in protein levels, whereas inhibition of autophagy is expected to be associated with protein accumulation as seen in studies performed on cell lines (60). To the interpretation of this result, it is possible to refer to two facts. Firstly, in the case of SQSTM1/p62, several transcription factors, including AP-1, NF-κB, Ets-1, and SP-1, have binding sites located in the 5'-flanking region of its promoter (61), as well as, its protein's structure is made up of several important domains that allow it to interact with crucial elements in vital signaling pathways. (59). Hence, p62 serves as both an autophagic cargo receptor and a major signaling hub that connects a number of crucial pro- and anti-inflammatory pathways in this instance (62). Secondly, HCQ is not just an autophagy inhibitor. It has been emphasized that the interpretation of the results of blocking autophagy requires caution because HCQ and CQ can cause multiple cellular changes (63). In this context, the results of the present study showed that HCQ therapy is accompanied by a decrease in SQSTM1/p62 gene expression. It is possible that since a prolonged treatment with HCQ was used in this study rather than cell studies, the contribution of the transcription downregulation in the cellular p62 pool could be more pronounced than the accumulation due to an acute inhibition of autophagy. Here, the reduced SQSTM1/p62 transcription could be in response to the general inflammation suppression by HCQ. Therefore, it may be better to consider SQSTM1/p62 transcriptional activity along with its protein expression to determine autophagy activity. For example, a ratio of these two could be more efficient and correct, although more studies are needed.

ER stress can trigger autophagy in mammalian cells, helping to remove misfolded proteins and damaged ER components, reducing ER burden, and promoting cell survival (64, 65). This study also demonstrated a connection between elevated ER stress and decreased autophagic flux in C57BL/6/J fed on HFD. Lastly, to bolster our theory that FSN-induced enhanced autophagy can impede the advancement of NAFLD caused by ER stress, Feng et al. have shown that GRP78 upregulates autophagy via AMPK phosphorylation and mTOR inhibition. In contrast, the autophagy inducer rapamycin alleviates ER stress to support cell survival (66). Consistently, our study confirmed a direct link between p-AMPK levels and the inactivation of mTOR gene transcription, leading to a decrease in GRP78 expression.

The findings of our study have raised one query: why, in spite of autophagy inhibition, does the FSN+HCQ group exhibit greater improvement than the FSN group? To address this question, further research is needed, particularly to examine the synergistic implications of FSN and HCQ on metabolic dysfunction. Interpreting the data in animal research is more complicated than in cell culture investigation to ascertain the part of any underlying mechanism. Still, it also gives us a more comprehensive and insightful perspective. In light of the aforementioned, it should be considered that ER dysfunction and immunometabolism signaling are integrated into multiple vicious cycles (67). This complexity increases in such a study that the hepatocytes are affected by a dynamic environment of the whole body.

In summary, we presented new research demonstrating that HFD-induced autophagic impairment was associated with hepatic lipid accumulation and ER stress burden, while FSN- treated group had an enhanced autophagic flux along with lowered lipid accumulation and ER stress intensity. Hence, these results suggest that FSN treatment prevented the lipid accumulation in HFD-fed mice, by restoring hepatic autophagy and subsequently reducing the ER stress.

**COMMITMENT TO RESEARCH INTEGRITY**

We hereby confirm that the study has been reported in accordance with the ARRIVE guidelines, ensuring transparency and completeness in the reporting of our research methods, results, and conclusions.

**AUTHOR CONTRIBUTIONS STATEMENT**

Mahboobe Sattari: performed the experiments, collected the data, and wrote the manuscript; Mohammad Esmaeil Shahaboddin: directed the project; Maryam Akhavan Taheri: carried out the histopathological evaluation; Ehsan Khalili: critically revised the manuscript for important intellectual content; Golnaz Goodarzi and Sadra Samavarchi Tehrani: contributed to data collection and analysis; Ozra Tabatabaei-Malazy: helped to funding acquisition; Reza Meshkani: contributed to the conception and design of the research; Ghodratollah Panahi: contributed to the conception and design of the research, reviewed and edited the manuscript, and had primary responsibility for the final content.

**COMPETING INTERESTS STATEMENT**

The authors declare no competing interests.

**DATA AVAILABILITY STATEMENT**

The data that support the findings of this study are available from the corresponding author upon reasonable request.

**REFERENCES**

1.	Kistler KD, Brunt EM, Clark JM, Diehl AM, Sallis JF, Schwimmer JB. Physical activity recommendations, exercise intensity, and histological severity of nonalcoholic fatty liver disease. The American journal of gastroenterology. 2011;106(3):460-8; quiz 9.

2.	Rinella ME. Nonalcoholic fatty liver disease: a systematic review. Jama. 2015;313(22):2263-73.

3.	Hernandez-Rodas MC, Valenzuela R, Videla LA. Relevant Aspects of Nutritional and Dietary Interventions in Non-Alcoholic Fatty Liver Disease. International journal of molecular sciences. 2015;16(10):25168-98.

4.	Jung UJ, Choi M-S. Obesity and its metabolic complications: the role of adipokines and the relationship between obesity, inflammation, insulin resistance, dyslipidemia and nonalcoholic fatty liver disease. International journal of molecular sciences. 2014;15(4):6184-223.

5.	Bessone F, Razori MV, Roma MG. Molecular pathways of nonalcoholic fatty liver disease development and progression. Cellular and molecular life sciences : CMLS. 2019;76(1):99-128.

6.	Rolo AP, Teodoro JS, Palmeira CM. Role of oxidative stress in the pathogenesis of nonalcoholic steatohepatitis. Free radical biology & medicine. 2012;52(1):59-69.

7.	Malhi H, Kaufman RJ. Endoplasmic reticulum stress in liver disease. Journal of hepatology. 2011;54(4):795-809.

8.	Puri P, Mirshahi F, Cheung O, Natarajan R, Maher JW, Kellum JM, et al. Activation and dysregulation of the unfolded protein response in nonalcoholic fatty liver disease. Gastroenterology. 2008;134(2):568-76.

9.	Pagliassotti MJ. Endoplasmic reticulum stress in nonalcoholic fatty liver disease. Annual review of nutrition. 2012;32:17-33.

10.	Vargas JNS, Hamasaki M, Kawabata T, Youle RJ, Yoshimori T. The mechanisms and roles of selective autophagy in mammals. Nature reviews Molecular cell biology. 2023;24(3):167-85.

11.	Fukuo Y, Yamashina S, Sonoue H, Arakawa A, Nakadera E, Aoyama T, et al. Abnormality of autophagic function and cathepsin expression in the liver from patients with non-alcoholic fatty liver disease. Hepatology research : the official journal of the Japan Society of Hepatology. 2014;44(9):1026-36.

12.	Fukushima H, Yamashina S, Arakawa A, Taniguchi G, Aoyama T, Uchiyama A, et al. Formation of p62-positive inclusion body is associated with macrophage polarization in non-alcoholic fatty liver disease. Hepatology research : the official journal of the Japan Society of Hepatology. 2018;48(9):757-67.

13.	Lavallard VJ, Gual P. Autophagy and non-alcoholic fatty liver disease. BioMed research international. 2014;2014:120179.

14.	Koga H, Kaushik S, Cuervo AM. Altered lipid content inhibits autophagic vesicular fusion. FASEB journal : official publication of the Federation of American Societies for Experimental Biology. 2010;24(8):3052-65.

15.	Yang L, Li P, Fu S, Calay ES, Hotamisligil GS. Defective hepatic autophagy in obesity promotes ER stress and causes insulin resistance. Cell metabolism. 2010;11(6):467-78.

16.	Singh R. Autophagy and regulation of lipid metabolism. Results and problems in cell differentiation. 2010;52:35-46.

17.	Kim Y, Lee DH, Park SH, Jeon TI, Jung CH. The interplay of microRNAs and transcription factors in autophagy regulation in nonalcoholic fatty liver disease. Experimental & molecular medicine. 2021;53(4):548-59.

18.	Akhlaghi M. Non-alcoholic Fatty Liver Disease: Beneficial Effects of Flavonoids. Phytotherapy research : PTR. 2016;30(10):1559-71.

19.	Xu MX, Ge CX, Qin YT, Gu TT, Lou DS, Li Q, et al. Multicombination Approach Suppresses Listeria monocytogenes-Induced Septicemia-Associated Acute Hepatic Failure: The Role of iRhom2 Signaling. Advanced healthcare materials. 2018;7(17):e1800427.

20.	Maher P, Dargusch R, Ehren JL, Okada S, Sharma K, Schubert D. Fisetin lowers methylglyoxal dependent protein glycation and limits the complications of diabetes. PloS one. 2011;6(6):e21226.

21.	Xu J, Yang KC, Go NE, Colborne S, Ho CJ, Hosseini-Beheshti E, et al. Chloroquine treatment induces secretion of autophagy-related proteins and inclusion of Atg8-family proteins in distinct extracellular vesicle populations. Autophagy. 2022;18(11):2547-60.

22.	Recena Aydos L, Aparecida do Amaral L, Serafim de Souza R, Jacobowski AC, Freitas Dos Santos E, Rodrigues Macedo ML. Nonalcoholic Fatty Liver Disease Induced by High-Fat Diet in C57bl/6 Models. Nutrients. 2019;11(12).

23.	Kim D-H, Do M-S. BAFF knockout improves systemic inflammation via regulating adipose tissue distribution in high-fat diet-induced obesity. Experimental & molecular medicine. 2015;47(1):e129-e.

24.	Melnyk RB, Boshes M. Efficiency of food utilization during body weight gain in dormice (Glis glis). Physiology & Behavior. 1980;24(6):1017-21.

25.	Benedé-Ubieto R, Estévez-Vázquez O, Ramadori P, Cubero FJ, Nevzorova YA. Guidelines and Considerations for Metabolic Tolerance Tests in Mice. Diabetes, metabolic syndrome and obesity : targets and therapy. 2020;13:439-50.

26.	Lai J, Wang HL, Zhang X, Wang H, Liu X. Pathologic Diagnosis of Nonalcoholic Fatty Liver Disease. Archives of pathology & laboratory medicine. 2022;146(8):940-6.

27.	Kielkopf CL, Bauer W, Urbatsch IL. Bradford Assay for Determining Protein Concentration. Cold Spring Harbor protocols. 2020;2020(4):102269.

28.	Zhang Z, Singh R, Aschner M. Methods for the detection of autophagy in mammalian cells. Current protocols in toxicology. 2016;69(1):20.12. 1-20.12. 26.

29.	Tian Y, Ma J, Wang W, Zhang L, Xu J, Wang K, et al. Resveratrol supplement inhibited the NF-κB inflammation pathway through activating AMPKα-SIRT1 pathway in mice with fatty liver. Molecular and cellular biochemistry. 2016;422(1-2):75-84.

30.	Liu L, Gao C, Yao P, Gong Z. Quercetin Alleviates High-Fat Diet-Induced Oxidized Low-Density Lipoprotein Accumulation in the Liver: Implication for Autophagy Regulation. BioMed research international. 2015;2015:607531.

31.	Xu M, Ge C, Qin Y, Gu T, Lv J, Wang S, et al. Activated TNF-α/RIPK3 signaling is involved in prolonged high fat diet-stimulated hepatic inflammation and lipid accumulation: inhibition by dietary fisetin intervention. Food & function. 2019;10(3):1302-16.

32.	Gaballah HH, El-Horany HE, Helal DS. Mitigative effects of the bioactive flavonol fisetin on high-fat/high-sucrose induced nonalcoholic fatty liver disease in rats. Journal of cellular biochemistry. 2019;120(8):12762-74.

33.	Correia da Silva D, Jervis PJ, Martins JA, Valentão P, Ferreira PMT, Pereira DM. Fisetin derivatives exhibit enhanced anti-inflammatory activity and modulation of endoplasmic reticulum stress. International immunopharmacology. 2023;119:110178.

34.	Ren H, Zhai W, Lu X, Wang G. The Cross-Links of Endoplasmic Reticulum Stress, Autophagy, and Neurodegeneration in Parkinson’s Disease. Frontiers in Aging Neuroscience. 2021;13.

35.	Liou CJ, Wei CH, Chen YL, Cheng CY, Wang CL, Huang WC. Fisetin Protects Against Hepatic Steatosis Through Regulation of the Sirt1/AMPK and Fatty Acid β-Oxidation Signaling Pathway in High-Fat Diet-Induced Obese Mice. Cellular physiology and biochemistry : international journal of experimental cellular physiology, biochemistry, and pharmacology. 2018;49(5):1870-84.

36.	Costa DK, Huckestein BR, Edmunds LR, Petersen MC, Nasiri A, Butrico GM, et al. Reduced intestinal lipid absorption and body weight-independent improvements in insulin sensitivity in high-fat diet-fed Park2 knockout mice. American journal of physiology Endocrinology and metabolism. 2016;311(1):E105-16.

37.	Fan Z, Xiao Q. Impaired autophagic flux contributes to muscle atrophy in obesity by affecting muscle degradation and regeneration. Biochemical and biophysical research communications. 2020;525(2):462-8.

38.	Cho Y, Chung JH, Do HJ, Jeon HJ, Jin T, Shin MJ. Effects of fisetin supplementation on hepatic lipogenesis and glucose metabolism in Sprague-Dawley rats fed on a high fat diet. Food chemistry. 2013;139(1-4):720-7.

39.	Kammoun HL, Chabanon H, Hainault I, Luquet S, Magnan C, Koike T, et al. GRP78 expression inhibits insulin and ER stress-induced SREBP-1c activation and reduces hepatic steatosis in mice. The Journal of clinical investigation. 2009;119(5):1201-15.

40.	Kim AJ, Shi Y, Austin RC, Werstuck GH. Valproate protects cells from ER stress-induced lipid accumulation and apoptosis by inhibiting glycogen synthase kinase-3. Journal of cell science. 2005;118(Pt 1):89-99.

41.	Cnop M, Foufelle F, Velloso LA. Endoplasmic reticulum stress, obesity and diabetes. Trends in molecular medicine. 2012;18(1):59-68.

42.	Nasiri-Ansari N, Nikolopoulou C, Papoutsi K, Kyrou I, Mantzoros CS, Kyriakopoulos G, et al. Empagliflozin Attenuates Non-Alcoholic Fatty Liver Disease (NAFLD) in High Fat Diet Fed ApoE((-/-)) Mice by Activating Autophagy and Reducing ER Stress and Apoptosis. International journal of molecular sciences. 2021;22(2).

43.	Cui W, Ma J, Wang X, Yang W, Zhang J, Ji Q. Free fatty acid induces endoplasmic reticulum stress and apoptosis of β-cells by Ca2+/calpain-2 pathways. PLoS One. 2013;8(3):e59921.

44.	Zhang Y, Xue R, Zhang Z, Yang X, Shi H. Palmitic and linoleic acids induce ER stress and apoptosis in hepatoma cells. Lipids in health and disease. 2012;11:1.

45.	Dai X, Kuang Q, Sun Y, Xu M, Zhu L, Ge C, et al. Fisetin represses oxidative stress and mitochondrial dysfunction in NAFLD through suppressing GRP78-mediated endoplasmic reticulum (ER) stress. Journal of Functional Foods. 2022;90:104954.

46.	Jo H, Choe SS, Shin KC, Jang H, Lee JH, Seong JK, et al. Endoplasmic reticulum stress induces hepatic steatosis via increased expression of the hepatic very low-density lipoprotein receptor. Hepatology. 2013;57(4):1366-77.

47.	Wang S, Chen Z, Lam V, Han J, Hassler J, Finck BN, et al. IRE1α-XBP1s induces PDI expression to increase MTP activity for hepatic VLDL assembly and lipid homeostasis. Cell metabolism. 2012;16(4):473-86.

48.	Ren LP, Yu X, Song GY, Zhang P, Sun LN, Chen SC, et al. Impact of activating transcription factor 4 signaling on lipogenesis in HepG2 cells. Mol Med Rep. 2016;14(2):1649-58.

49.	Schott MB, Weller SG, Schulze RJ, Krueger EW, Drizyte-Miller K, Casey CA, et al. Lipid droplet size directs lipolysis and lipophagy catabolism in hepatocytes. The Journal of cell biology. 2019;218(10):3320-35.

50.	Niture S, Lin M, Rios-Colon L, Qi Q, Moore JT, Kumar D. Emerging Roles of Impaired Autophagy in Fatty Liver Disease and Hepatocellular Carcinoma. International journal of hepatology. 2021;2021:6675762.

51.	Alers S, Löffler AS, Wesselborg S, Stork B. Role of AMPK-mTOR-Ulk1/2 in the regulation of autophagy: cross talk, shortcuts, and feedbacks. Molecular and cellular biology. 2012;32(1):2-11.

52.	Kim YC, Guan KL. mTOR: a pharmacologic target for autophagy regulation. The Journal of clinical investigation. 2015;125(1):25-32.

53.	Liou C-J, Wei C-H, Chen Y-L, Cheng C-Y, Wang C-L, Huang W-C. Fisetin Protects Against Hepatic Steatosis Through Regulation of the Sirt1/AMPK and Fatty Acid β-Oxidation Signaling Pathway in High-Fat Diet-Induced Obese Mice. Cellular Physiology and Biochemistry. 2018;49(5):1870-84.

54.	Jung CH, Kim H, Ahn J, Jeon T-I, Lee D-H, Ha T-Y. Fisetin regulates obesity by targeting mTORC1 signaling. The Journal of Nutritional Biochemistry. 2013;24(8):1547-54.

55.	Syed DN, Chamcheu JC, Khan MI, Sechi M, Lall RK, Adhami VM, et al. Fisetin inhibits human melanoma cell growth through direct binding to p70S6K and mTOR: findings from 3-D melanoma skin equivalents and computational modeling. Biochemical pharmacology. 2014;89(3):349-60.

56.	Ding H, Li Y, Chen S, Wen Y, Zhang S, Luo E, et al. Fisetin ameliorates cognitive impairment by activating mitophagy and suppressing neuroinflammation in rats with sepsis-associated encephalopathy. CNS neuroscience & therapeutics. 2022;28(2):247-58.

57.	Zhang J, Zhao L, Hu C, Wang T, Lu J, Wu C, et al. Fisetin Prevents Acetaminophen-Induced Liver Injury by Promoting Autophagy. Frontiers in pharmacology. 2020;11:162.

58.	Kim S, Choi KJ, Cho SJ, Yun SM, Jeon JP, Koh YH, et al. Fisetin stimulates autophagic degradation of phosphorylated tau via the activation of TFEB and Nrf2 transcription factors. Scientific reports. 2016;6:24933.

59.	Puissant A, Fenouille N, Auberger P. When autophagy meets cancer through p62/SQSTM1. American journal of cancer research. 2012;2(4):397-413.

60.	He Q, Sha S, Sun L, Zhang J, Dong M. GLP-1 analogue improves hepatic lipid accumulation by inducing autophagy via AMPK/mTOR pathway. Biochemical and biophysical research communications. 2016;476(4):196-203.

61.	Vadlamudi RK, Shin J. Genomic structure and promoter analysis of the p62 gene encoding a non-proteasomal multiubiquitin chain binding protein. FEBS letters. 1998;435(2-3):138-42.

62.	Hennig P, Fenini G, Di Filippo M, Karakaya T, Beer HD. The Pathways Underlying the Multiple Roles of p62 in Inflammation and Cancer. Biomedicines. 2021;9(7).

63.	Mauthe M, Orhon I, Rocchi C, Zhou X, Luhr M, Hijlkema KJ, et al. Chloroquine inhibits autophagic flux by decreasing autophagosome-lysosome fusion. Autophagy. 2018;14(8):1435-55.

64.	Ogata M, Hino S, Saito A, Morikawa K, Kondo S, Kanemoto S, et al. Autophagy is activated for cell survival after endoplasmic reticulum stress. Molecular and cellular biology. 2006;26(24):9220-31.

65.	Kouroku Y, Fujita E, Tanida I, Ueno T, Isoai A, Kumagai H, et al. ER stress (PERK/eIF2alpha phosphorylation) mediates the polyglutamine-induced LC3 conversion, an essential step for autophagy formation. Cell death and differentiation. 2007;14(2):230-9.

66.	Feng J, Chen Y, Lu B, Sun X, Zhu H, Sun X. Autophagy activated via GRP78 to alleviate endoplasmic reticulum stress for cell survival in blue light-mediated damage of A2E-laden RPEs. BMC ophthalmology. 2019;19(1):249.

67.	Hotamisligil GS. Endoplasmic reticulum stress and the inflammatory basis of metabolic disease. Cell. 2010;140(6):900-17.

**Supporting information**

**S1 Table. Supplementary Table 1.** Primers sequences used in this study.
